# Beta-Caryophyllene Attenuates the In Vitro Oxidation of LDL

**DOI:** 10.3390/biomedicines14091938

**Published:** 2026-08-29

**Authors:** Gerhard Cvirn, Margret Paar, Christine Rossmann, Azra Darko, Gerd Kager, Gerhard Ledinski, Thomas Wagner, Seth Hallström, Gilbert Reibnegger, Tobias Ziegler, Willibald Wonisch

**Affiliations:** 1 Division of Medicinal Chemistry, Otto Loewi Research Centre, Medical University of Graz, 8010 Graz, Austria; gerhard.cvirn@medunigraz.at (G.C.); margret.paar@medunigraz.at (M.P.); christine.rossmann@medunigraz.at (C.R.); azra.darko@medunigraz.at (A.D.); gerd.kager@medunigraz.at (G.K.); gerhard.ledinski@medunigraz.at (G.L.); seth.hallstroem@medunigraz.at (S.H.); gilbert.reibnegger@medunigraz.at (G.R.); tobias.ziegler@stud.medunigraz.at (T.Z.); 2 Department of Blood Group Serology and Transfusion Medicine, Medical University of Graz, 8010 Graz, Austria; thomas.wagner@medunigraz.at; 3 Division of Biomedical Research and Translational Medicine, Medical University of Vienna, 1090 Vienna, Austria

**Keywords:** anti-oxidants, atherogenesis, copper ions, low-density lipoprotein, lipid oxidation, reactive oxygen species

## Abstract

**Background/Objectives**: The oxidation of low-density lipoprotein (LDL) is a crucial step in atherogenesis. Beta-Caryophyllene (BCP) is a natural compound with established anti-oxidative and anti-inflammatory properties as shown in animal and cell culture studies. We examined whether BCP can impede LDL oxidation in an in vitro model. **Methods**: The anti-oxidative effect of BCP was evaluated in different concentrations (0, 25, 50, 100, and 150 µg/mL) with regard to scavenging reactive oxygen species (ROS) during LDL oxidation, which was initiated by the addition of copper chloride (CuCl_2_) in a concentration of 10 µmol/L. Lipid hydroperoxides (LPO), malondialdehyde (MDA), dienes, cell viability, and reactions of BCP with ROS according to Gibbs free energies were applied to determine the oxidation state of LDL. **Results**: Our findings indicated that BCP is highly efficient in inhibiting LDL oxidation in a dose-dependent manner in this in vitro model. The lipid hydroperoxide content in oxLDL was significantly lower in the presence of 100 µg/mL BCP compared to oxLDL without BCP (*p* < 0.0001). This corresponds to the MDA levels, which were significantly lower in the presence of 100 µg/mL BCP compared to oxLDL without BCP (*p* = 0.0393). Furthermore, a dose-dependent inhibition of diene formation in the LDL particle was observed in the presence of ascending BCP concentrations which corresponds to a decrease in the cytotoxicity of oxLDL in EA.hy926 cells in the presence of increasing concentrations of BCP. Moreover, BCP’s anti-oxidant effectiveness exceeds that of the widely recognized anti-oxidant spermidine at equivalent concentrations. Our quantum chemical calculations showed that the reactions between BCP and hydroxyl radicals, hydroperoxyl radicals, or hydrogen peroxide are exergonic. We therefore conclude that BCP impedes the oxidation of LDL by its capability to scavenge (at least) these three reactive oxygen species. **Conclusions**: Our results indicate that BCP impedes the oxidation of LDL in vitro and therefore presumably has the potential to serve as an appropriate therapeutic agent to prevent atherogenesis and related (cardio)vascular diseases by balancing vascular oxidative stress. For this purpose, more prospective clinical studies in humans are required to assess the potential atheroprotective and health-promoting effects of BCP.

## 1. Introduction

Beta-Caryophyllene (BCP) is a natural sesquiterpene present in numerous herbs, spices, and foods [1]. BCP is widely used in cosmetics and as a safe food flavoring additive with approval from the Food and Drug Administration (FDA) and the European Food Safety Authority (EFSA) [2,3,4].

Numerous studies have shown that BCP possesses, e.g., anti-inflammatory [5], anti-angiogenic [6], anti-oxidative [7,8,9], anti-microbial [10], cannabinoid [11], anti-carcinogenic [12], skin-penetration-enhancing [13], anti-spasmodic [14], and local anesthetic properties [15].

Moreover, BCP has been reported to impede lipid peroxidation by virtue of its radical-scavenging activity against hydroxyl radicals (HO^•^), superoxide anions (O_2_^•−^), and lipid peroxides [1,16]. Its anti-oxidant capacity is apparently attributable to its chemical structure, including a bicyclic system with double rings [17].

Native LDL (nLDL) does not promote atherosclerosis. Nevertheless, in arterial lesions within the subendothelial space, it can, however, undergo oxidation through processes that involve reactive oxygen species (ROS) and/or lipoxygenases [18].

By attaching to scavenger receptors on smooth muscle cells and macrophages, oxidized low-density lipoprotein (oxLDL) promotes the development of foam cells. This causes cholesterol ester buildup, enormous, uncontrolled lipid uptake, and persistent inflammation, transforming healthy immune cells into the fat-filled “foam cells” that develop into early atherosclerotic plaques.

Among others, ROS have been shown to be generated by NADPH oxidase or uncoupled eNOS [19]. The oxidized form of LDL (oxLDL) exerts significant cytotoxic effects on endothelial cells, representing a key event in the course of atherosclerosis [20]. OxLDL has been shown to impair endothelial production of NO and to induce the expression of leukocyte adhesion molecules [21]. We deployed a well-established in vitro model for LDL oxidation by Cu^2+^ ions [22], which was quantified by the measurement of lipid hydroperoxides (LPO), malondialdehyde (MDA), as well as diene formation. Oxidation of the protein part of the LDL particle was quantified via oxidation-specific immune epitopes [23]. OxLDL cytotoxicity was assessed by the MTT viability test in the vascular endothelial cell line EA.hy926 [22,24]. Furthermore, the anti-oxidative action of BCP was compared with that of spermidine, a well-known anti-oxidant. In addition, quantum chemical calculations were applied to evaluate the anti-oxidative mechanisms of BCP to impede nLDL oxidation. In this model experiment, Cu^2+^ ions form initiating ROS, namely HO^•^, hydroperoxyl radicals (HOO^•^), O_2_^•−^, and hydrogen peroxide (H_2_O_2_) [25,26]. The respective negative values of Gibbs free reaction energy denote which ROS is likely to be scavenged by BCP.

The aim of this study was to investigate whether BCP is capable of impeding the oxidation of nLDL, a crucial feature to protect against the development of endothelial dysfunction, which is followed by atherogenesis and (cardio)vascular disease, as a potential anti-oxidative substance.

## 2. Materials and Methods

### 2.1. Preparation of nLDL

This study was approved by the ethics committee of the Medical University of Graz, and all participants provided written informed consent (27-320 ex 14/15). Human LDL (1.020 to 1.063 g/mL) was isolated from the plasma of four normolipemic (Lp(a) < 5 mg/dL), fasting (12 to 14 h) young male individuals using sequential ultracentrifugation with potassium bromide [20,22,27]. To avert lipid peroxidation and the cleavage of apolipoprotein B (apoB) by proteinases and potential contaminating bacteria, Pefabloc 50 µM (Sigma Aldrich, Vienna, Austria) and DTPA 100 µM (Sigma Aldrich, Vienna, Austria) were applied throughout all phases of lipoprotein preparation. The samples were sterile-filtered and kept in the dark at 4 °C until they were used. To assess the protein content of LDL, the Lowry method was utilized [28].

### 2.2. LDL Oxidation by the Use of Cu^2+^ Ions

nLDL (1.5 mg/mL) was incubated with increasing BCP concentrations (0–150 µg/mL, i.e., 0, 25, 50, 100, and 150 µg/mL) at 37 °C (pH = 7.4) in a 0.01 mol/L phosphate-buffered solution containing 0.154 mol/L NaCl. DTPA was completely removed by using PBS (0.01 M, pH = 7.4) and dialysis tubing (MWCO 14000, Membra-Cel cellulose, Carl Roth/Lactan, Graz, Austria). The oxidation of LDL was started with the addition of CuCl_2_ (final concentration of 10 µmol/L) during an incubation time of up to eight hours. As for cell experiments, the incubation with copper ions was terminated when LPO hit a threshold of 100 nmol/mg LDL without BCP (LDL controls = LDLc). Afterward, oxLDL underwent overnight dialysis at 4 °C using PBS.

### 2.3. Determination of Lipid Hydroperoxides (LPOs)

LPO levels in the corresponding oxLDLs were measured using a spectrophotometric assay for lipid hydroperoxides in serum lipoproteins [22,27]. The absorbance was recorded at 365 nm as demonstrated previously [22]. The molar absorptivity of I_3_, ε = 2.46 × 10^4^ M^−1^cm^−1^ was used to calculate the concentration. The calibration curves derived from various peroxides, including H_2_O_2_, t-butyl hydroperoxide, and cumene hydroperoxide, yielded ε values of 2.45 ± 0.04, 2.34 ± 0.26, and 1.26 ± 0.15 × 10^4^ M^−1^cm^−1^, respectively. A stoichiometric relationship (slope = 1.02) was noted between the quantity of organic peroxides tested and the concentration of I_3_ generated.

### 2.4. Determination of Malondialdehyde

MDA was measured using a high-performance liquid chromatography (HPLC) method described by Pilz et al. [29] following derivatization with 2,4-dinitrophenylhydrazine (DNPH). As previously described [29], protein-bound MDA was hydrolyzed and deproteinized. Thereafter, a volume of 12.5 µL from the DNPH solution was mixed with 125 µL of the supernatant and subsequently injected into the HPLC system (with an injection volume of 40 µL). The MDA standard was generated as depicted recently [29]. The isocratic separation of DNPH derivatives (hydrazones) was performed using HPLC, which included an L-2200 autosampler, L-2130 HTA pump, and L-2450 diode array detector (all: VWR Hitachi; Vienna; Austria). Signals from the detector (absorbance at 310 nm) were noted, and data acquisition and analysis were performed with EZchrom Elite software (VWR).

### 2.5. Determination of Oxidation-Specific Immune Epitopes

The formation of oxidation-induced epitopes on apoB was monitored using a monoclonal antibody directed against modified apoB with a solid phase dissociation-enhanced lanthanide fluorescence immunoassay (DELFIA^®^, Revvity Health Sciences, Turku, Finland), as previously described [23]. Anti-ox-apoB (OB 04) is a monoclonal antibody developed against copper-oxidized LDL, known for its specific reaction with oxidized apoB-containing lipoproteins [23]. Another antibody, i.e., a rabbit polyclonal antibody (anti-apoB), was purchased from Behring (Marburg, Germany). Both antibodies were applied to detect respective antigens. In case of OB 04, Eu^3+^-labeled rabbit anti-mouse IgG (for OB/04) was used compared to Eu^3+^-labeled sheep anti-rabbit IgG in case of anti-apoB. The quantity of oxidation-specific epitopes on the LDL particle was represented as the ratio of oxidatively modified LDL counts to nLDL counts, as detailed in previous studies [22,30].

### 2.6. Cell Culture

EA.hy926 cells from the American Type Culture Collection (ATCC) were kindly provided by Dr. C.J. Edgell (University of North Carolina, Chapel Hill, NC, USA) [31]. EA.hy926 cells were generated by fusing primary human umbilical vein endothelial cells with a thioguanine-resistant clone of the A549 cell line [32]. EA.hy926 cells have been demonstrated to exhibit a similar behavior to that of primary endothelial cells in numerous aspects [33,34]. These EA.hy926 cells were cultured as described previously [22]. nLDL and oxLDL (oxidized with Cu^2+^ ions) were incubated in the presence of ascending BCP concentrations and used for the cell experiment at a concentration of 0.4 mg/mL for as long as 8 h in EA.hy926 cells.

### 2.7. Cell Viability (MTT Test)

The MTT (3-(4,5-dimethyl-2-thiazolyl)-2,5-diphenyl-2H-tetrazolium bromide) assay was used to assess the metabolic activity of EA.hy926 cells treated with Cu^2+^-oxidized LDL. Cells grown to confluence in 12- or 24-well plates were treated with the respective lipoproteins (with or without BCP) at a concentration of 0.3 mg/mL for the specified durations (2 to 8 h). To evaluate the cell viability, these cells were further treated with MTT (1.2 mM in serum-free medium) and incubated for 2 h at 37 °C under standard conditions. Cells were washed using PBS and lysed with isopropanol/1 M HCl (25:1; *v*/*v*) on a microplate shaker at 1200 rpm for 15 min. Absorbance measurements were performed at 570 nm using a Power Wave X Select microplate spectrophotometer (BioTek Germany, Bad Friedrichshall, Germany) and adjusted for background absorption at 650 nm.

### 2.8. Quantum Chemical Calculations for the Anti-Oxidative Effect Against ROS by BCP

Becke’s 3-parametric density exchange functional, combined with the correlation function by Lee, Yang and Parr, was employed for density functional theory (DFT) calculations (restricted for neutral species, unrestricted for radical species). All calculations involved geometry optimizations and vibrational analyses to ascertain the thermal contributions to free energies. The basis set 6–311+ G (2d,p) was used to perform the calculations. The calculations were performed by simulating a polar (water) environment, using the SMD model [35]. The changes in Gibb’s free energies (delta G^0^-values) were calculated for the reactions examined under standard conditions (298.15 K, 101.325 kPa). Electron densities, electrostatic potentials, and—for open shell systems—spin density distributions were obtained for all examined molecular structures. All quantum chemical calculations and result analyses were performed using the GAUSSIAN suite of quantum chemistry programs (GaussView 6.0 and Gaussian G16 W, Gaussian Inc., Pittsburgh, PA, USA).

### 2.9. Statistics

The GraphPad Prism Package (v. 10) was used for statistical evaluation. The Shapiro–Wilk test was applied to investigate if the variables were normally distributed. Differences between mean values of variables were tested for statistical differences by repeated measures ANOVA followed by Dunnett’s multiple comparisons test (in case of normally distributed variables) or by the non-parametric Friedman test followed by Dunn’s multiple comparisons test (in case of not normally distributed variables). *p* values less than 0.05 were considered statistically significant: * *p* ≤ 0.05, ** *p* ≤ 0.01, *** *p* ≤ 0.001, and **** *p* ≤ 0.0001.

## 3. Results

### 3.1. Effect of BCP on the Oxidizability of the Lipid Part of the LDL Particle

**Figure 1:**
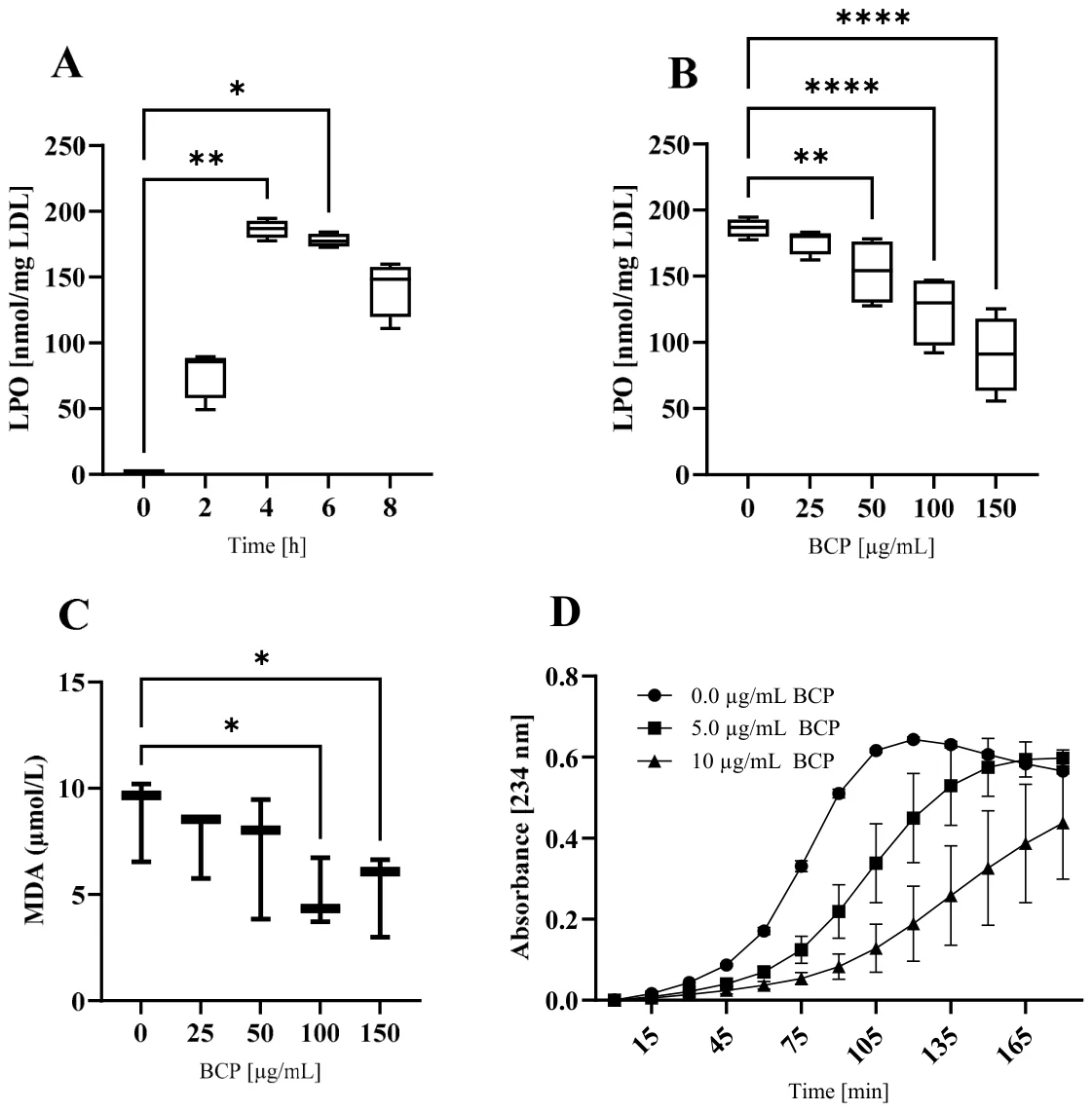
Oxidation of the lipid component of LDL. Time-course of lipid hydroperoxides (LPO) formation (**A**), suppression of LPO formation by BCP (**B**), suppression of malondialdehyde (MDA) formation by BCP (**C**), suppression of diene formation by BCP (**D**). * *p* ≤ 0.05, ** *p* ≤ 0.01, and **** *p* ≤ 0.0001.

nLDL preparations were oxidized by the addition of CuCl_2_ (10 µmol/L) for up to 8 h in the presence of 0, 25, 50, 100, or 150 µg/mL of BCP. The respective LPO content of LDL, which indicates the oxidative status of the lipid part of the LDL particle, increased in a time-dependent manner and reached a maximum between four and six hours of incubation, comparable to the findings of a previous study [29], as shown in Figure 1A.

BCP suppressed the Cu^2+^-induced oxidation in the lipid component of LDL in a concentration-dependent manner, as indicated by lipid peroxidation biomarkers; i.e., the formation of LPO and MDA decreased with increasing concentrations of BCP, as shown in Figure 1B and Figure 1C, respectively. Moreover, diene formation concentration-dependently decreased in the presence of distinct lower concentrations (5 and 10 µg/mL) of BCP, as shown in Figure 1D.

LDL (1.5 mg/mL) was preincubated with increasing amounts of BCP (0, 25, 50, 100, and 150 µg/mL) and then, in a second step, oxidized with CuCl_2_ (10 µmol/L) for up to 8 h. Panel A shows the formation of lipid hydroperoxides (LPO) during Cu^2+^-triggered oxidation of LDL. Experiments were conducted four times in LDL from a single donor. In the absence of BCP, the lipid hydroperoxide (LPO) content of LDL increased time-dependently (*p* < 0.0001, Friedman test). Panel B shows the influence of BCP on LPO formation during Cu^2+^-triggered oxidation of LDL. BCP suppressed the formation of LPO during an oxidation procedure for 4 h, concentration-dependently, as well (*p* < 0.0001, RM one-way ANOVA). The data represent the median (min-max) of four measurements conducted in LDL from a single donor. Panel C shows the influence of BCP on MDA formation during Cu^2+^-triggered oxidation of LDL. BCP concentration-dependently suppressed the formation of malondialdehyde (MDA) during LDL oxidation for 2 h, as well (*p* = 0.0028, Friedman test). The data represent the median (min-max) of three measurements conducted with LDL from a single donor. This chain of evidence was also supported, as BCP suppressed the diene formation in a concentration-dependent manner. The data represent mean ± SD from four separate measurements performed with LDL from a single donor, shown in panel D.

### 3.2. Effect of BCP on the Oxidizability of the Protein Component of the LDL Particle

**Figure 2:**
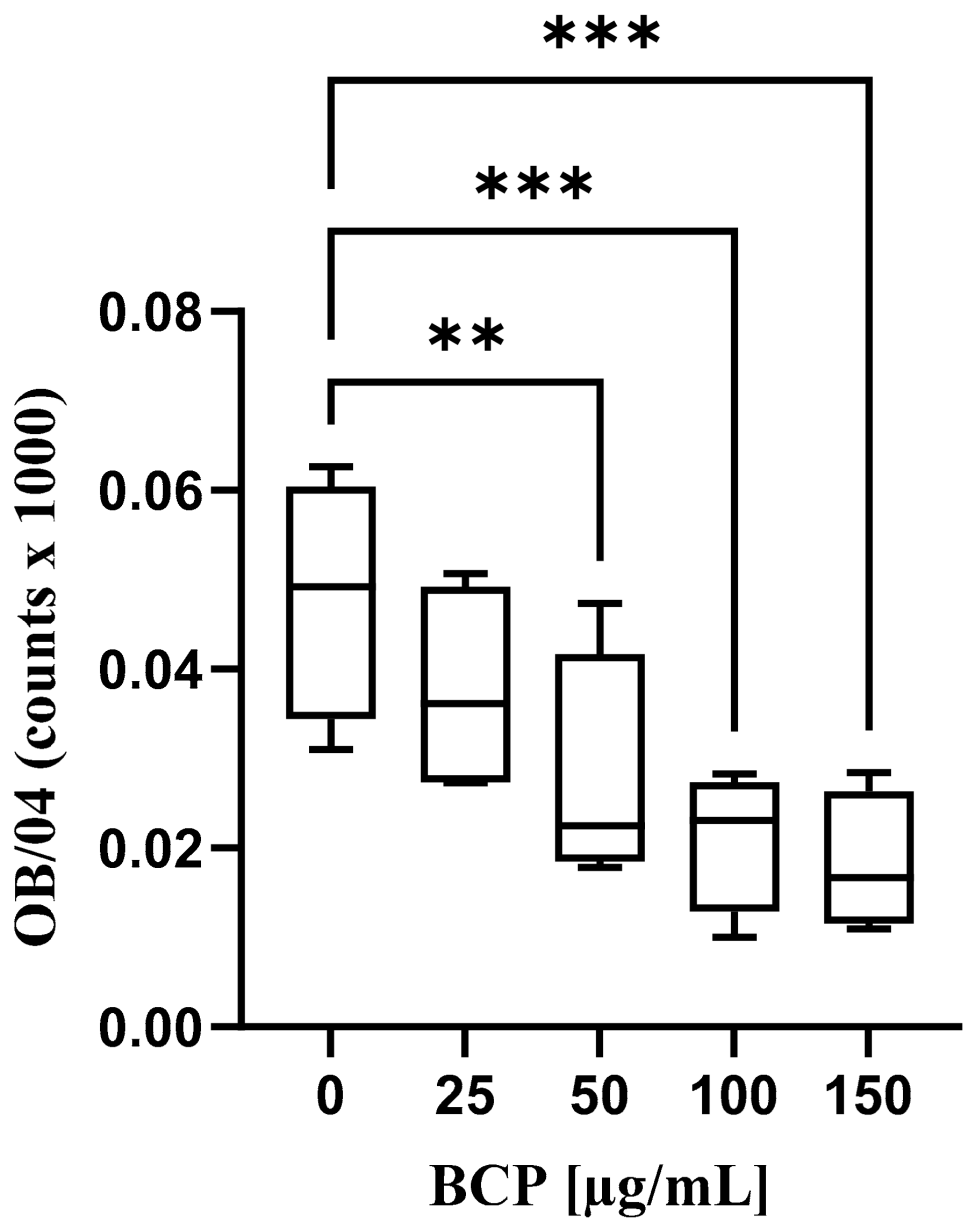
Cu^2+^-induced oxidation of the protein component of LDL. ** *p* ≤ 0.01, *** *p* ≤ 0.001.

In the course of the oxidation procedure, the amount of oxidation-specific epitopes decreased concentration-dependently in the presence of increasing BCP concentrations, as shown in Figure 2, showing that BCP is capable of suppressing the oxidation of the LDL protein component.

LDL (1.5 mg/mL) was preincubated with increasing concentrations of BCP and then oxidized with CuCl_2_ (10 µmol/L) for up to 8 h. BCP concentration-dependently suppressed the formation of oxidation-specific epitopes when LDL was oxidized with Cu^2+^ ions for 4 h (*p* < 0.0001, RM one-way ANOVA), as indicated by a significantly lower monoclonal antibody binding. The data represent the median (min-max) from four separate measurements.

### 3.3. Anti-Oxidative Action of BCP Compared to Spermidine

**Figure 3:**
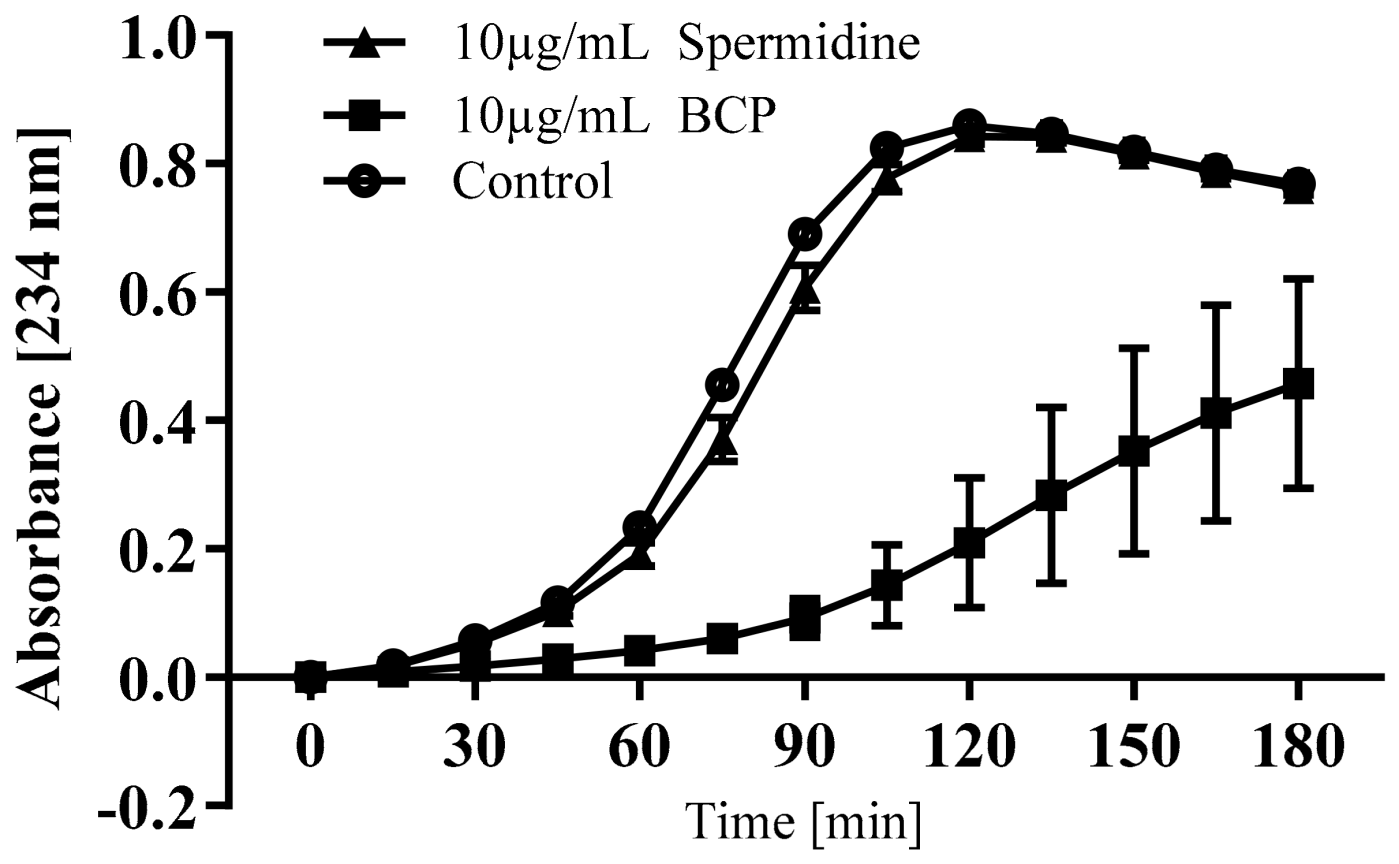
Suppression of LDL oxidation by BCP compared with spermidine.

Cu^2+^-induced diene formation in LDL was suppressed concentration-dependently by spermidine, as shown in Figure 3. The same effect, albeit much more pronounced, was observed with equimolar concentrations of BCP during LDL oxidation.

Diene formation in the absence of anti-oxidants (spheres) and in the presence of 10 µg/mL spermidine (triangles) or 10 µg/mL BCP (squares). The data represent mean ± SD from three separate measurements.

### 3.4. Decreasing Cytotoxicity of oxLDL in the Presence of Increasing Concentrations of BCP in EA.hy926 Cells

**Figure 4:**
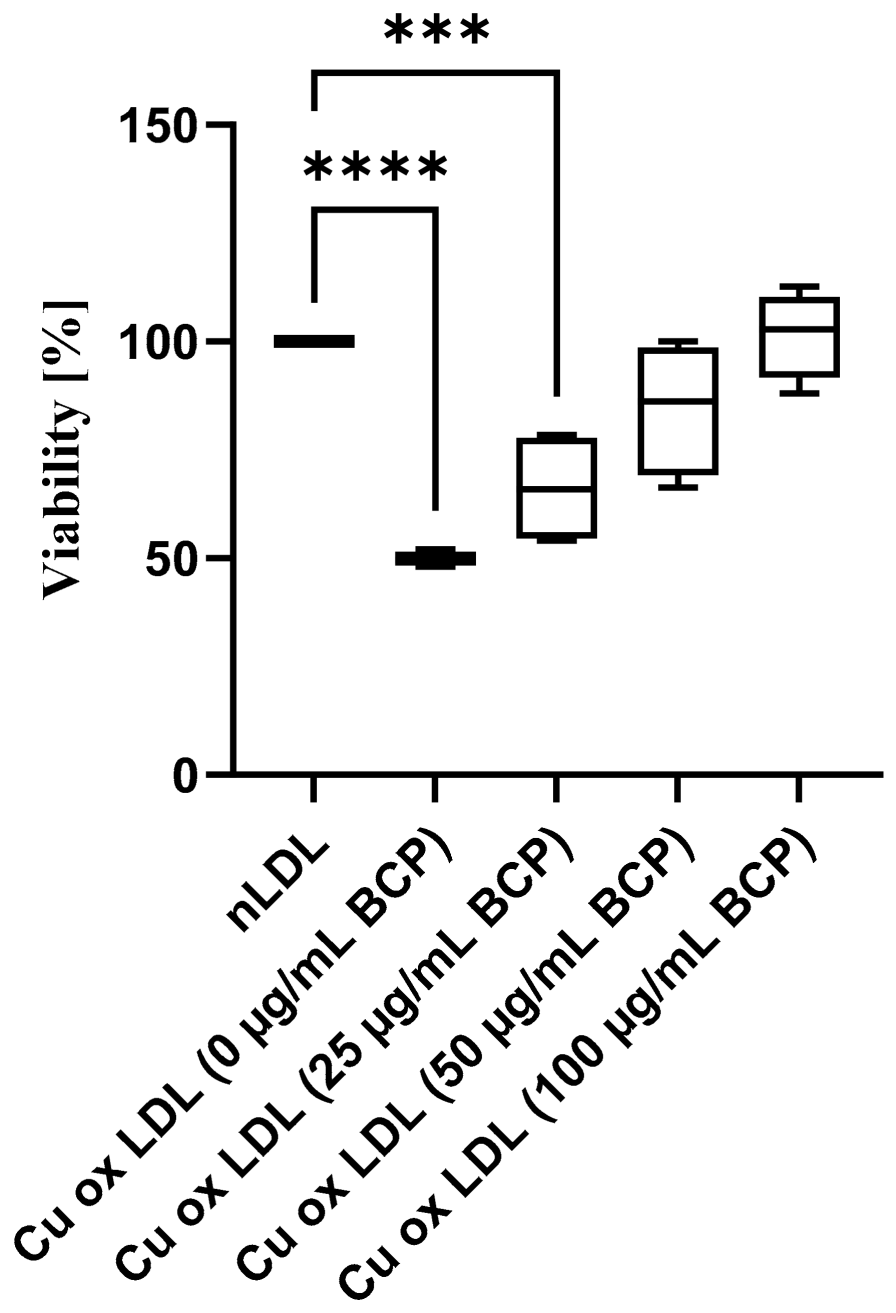
Effect of BCP on the cell viability of oxLDL in EAhy.926 cells. *** *p* ≤ 0.001, and **** *p* ≤ 0.0001.

The cytotoxicity of oxLDL decreased concentration-dependently in EA.hy926 cells when it was generated in the presence of increasing concentrations of BCA, as shown in Figure 4. Cell viability was significantly reduced in oxLDL-incubated cells (*p* < 0.0001, RM one-way ANOVA), but was restored when oxLDL was formed in the presence of BCP.

LDL was preincubated with 0, 25, 50, and 100 µg/mL BCP and subsequently oxidized with Cu^2+^ ions until LPO levels reached 100 nmol/mg LDL protein. Cell viability decreased significantly in the presence of oxLDL but was transiently recovered when the oxidation procedure was performed in the presence of 50 and 100 µg/mL of BCP. The data represent the median (min-max) from four separate measurements. Cell viability for native LDL was set at 100% for each experiment.

### 3.5. Reactions of BCP with Hydroxyl Radicals, Hydroperoxyl Radicals, Superoxide Radical Anions, or Hydrogen Peroxide

Gibbs free energies for the abstraction of one hydrogen atom from BCP by the hydroxyl radical (to yield water), by the hydroperoxyl radical (to yield hydrogen peroxide), or by the superoxide radical anion (to yield hydroperoxide anion) were calculated and are shown in Table 1.

**Figure 5:**
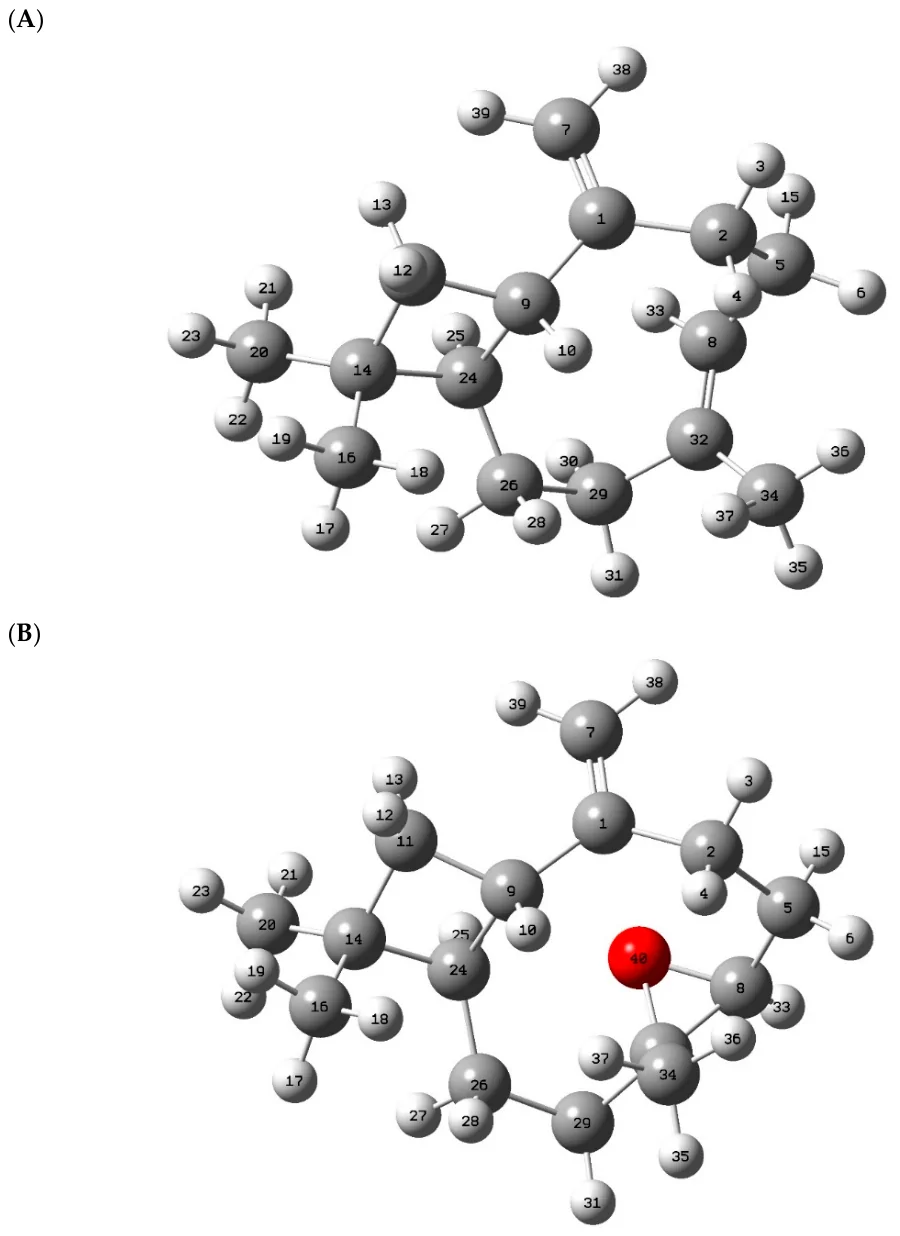
Chemical structure of BCP (**A**) and BCP epoxide (**B**), respectively. Small spheres: hydrogen; large light spheres: carbon; large dark sphere: oxygen.

The abstraction of hydrogens (numbers 3, 10, 15, 25, and 30) from BCP were studied according to the scheme shown in Figure 5A.

The reactions of BCP with HO^•^ and partly with HOO^•^ were exergonic. This is shown by the negative values for the respective Gibbs free energies, whereby the abstraction of hydrogen atoms by HO^•^ resulted in the highest negative energy values. Therefore, BCP may protect nLDL from oxidative modification. Reactions of O_2_^•−^ with BCP were endergonic (positive values of the respective Gibb free energies), and, therefore, are unlikely to occur spontaneously. Moreover, our quantum chemical calculations for the reaction between BCP and hydrogen peroxide (to form BCP epoxide and water) yielded a Gibbs free energy of −7.23 kcal/mol, suggesting that this reaction is thermodynamically exergonic, and, thus, could occur spontaneously. The chemical structure of BCP epoxide is shown in Figure 5B. In summary, our quantum chemical calculations suggest that BCP exerts its anti-oxidative action by neutralizing at least the ROS HO^•^, HOO^•^, and H_2_O_2_.

## 4. Discussion

In this study we demonstrate that the natural sesquiterpene BCP may protect the oxidation of nLDL to a certain degree. Thus, this represents an essential prevention in the onset of endothelial dysfunction and atherogenesis [36,37,38].

We used an in vitro model where nLDL is oxidized through the addition of Cu^2+^ ions [29]. The main finding of this study is that BCP reduces the oxidation of both the lipid and the protein components of the LDL particle in a concentration-dependent manner, at concentrations significantly higher than those found in plasma. In line with this, the cytotoxicity of oxLDL in EAhy.926 cells decreased when it was generated at higher BCP concentrations.

Furthermore, we demonstrate the suppression of diene formation in the LDL particle, where BCP’s anti-oxidative effect surpasses that of the widely recognized anti-oxidant spermidine several-fold, at equivalent concentrations.

As the processes by which Cu^2+^ triggers nLDL oxidation are not fully understood, we can only speculate about the mechanism in which BCP prevents nLDL oxidation in our model experiment. Previous research indicates that at least four specific ROS are involved in LDL oxidation: HO^•^, HOO^•^, O_2_^•−^, and H_2_O_2_ [18,39]. At least three of these ROS may react with BCP under our experimental conditions. Based on our calculations of reaction free energies, we assume that the hydroxyl radical is the most probable candidate for BCP scavenging, but also the hydroperoxyl radical as well as hydrogen peroxide might spontaneously react with BCP.

This study confirmed the findings of Calleja et al., who have shown high scavenging activities of BCP against hydroxyl radicals [1]. However, in their experiments, BCP also showed scavenging activity against the superoxide anion. This is in contrast to our quantum chemical calculations which showed that the reaction between the superoxide anion and BCP is endergonic. This discrepancy might be attributable to the different experimental conditions since Callja et al. used the Riboflavin assay to examine the scavenging activity of BCP. They also showed that BCP, under their experimental conditions, had a higher inhibiting capacity on lipid peroxidation than probucol, alpha-humullene, and alpha-tocopherol [1]. Potent in vitro scavenging activity of BCP against the two radicals mentioned above was also reported by Al-Taee et al. [40]. Yovas et al. have shown in an in vitro experiment that BCP is capable of scavenging further free radicals, namely 1, 1-diphenyl-2-picrylhydrazyl radicals (DPPH^•^) and azino-bis-(3-ethylbenzothiazoline-6-sulfonic acid) radicals (ABTS^•+^) in a concentration-dependent manner [41]. The structure of BCP has been suggested to allow radical insertion on the ring. Therefore, Ojha et al. concluded that BCP is capable of reacting with peroxyl, hydroxyl, and superoxide anions [42].

The ability of BCP to inhibit the oxidation of nLDL in vivo could be due to BCP being apparently capable of penetrating lipophilic compartments. Scandiffio et al. have shown that BCP is able to cross plasma membranes [43]. Thus, BCP seems to penetrate both, i.e., the subendothelial space of arteries, a highly pro-oxidative milieu and the LDL particle itself, where it might exert its anti-oxidant properties. Due to its lipophilicity, BCP is able to diffuse into the lipid-rich region in the core of the LDL particle to act there as an anti-oxidant agent [22].

The main finding of this study is the capability of BCP to attenuate, at least in part, ROS-induced endothelial-dysfunction-associated atherogenesis and (cardio)vascular damage through its anti-oxidant activity, which impedes nLDL oxidation. Hence, inhibition of oxLDL might prevent the development of succeeding atherosclerosis, e.g., by the diminished existence of oxLDL, a defense mechanism which prevents downstream operations.

However, clinical research on the effects of BCP in humans is sparse, which renders therapeutic claims premature. Thus, the main evidence for BCP effects remains on animal and cell culture studies. For example, Younis et al. have shown that BCP is a protective agent against myocardial injury in rats [44].

Thus, our present study serves as an impetus for future clinical trials on the effects of BCP in humans. BCP apparently possesses vast anti-oxidant potential for numerous diseases linked to inflammation and oxidative stress, besides atherogenesis, meriting further investigation [3].

Since BCP acts only on cannabinoid receptor type 2 (CB2) and not on CB1 receptors, it can be regarded as a safe and non-psychoactive phytocannabinoid [43]. Moreover, BCP shows beneficial effects on obesity and on non-alcoholic fatty liver disease [45] and has been shown to display neuroprotective activity in animal models [9,46]. Therefore, future studies are urgently warranted to test whether these findings can be reproduced in human subjects.

However, possible adverse effects have to be taken into account, especially when using BCP in higher concentrations. BCP at 300 mg/kg can cause liver necrosis, liver weight gain, and bile duct disorders in rats [16]. Furthermore, it has to be considered that BCP might interact with drugs and other food components. BCP has been shown to inhibit enzymes involved in xenobiotic detoxification [47]. This effect could result in prolonged duration of action and increased toxicity of these molecules.

Moreover, possible gender differences must be taken into account in future clinical studies. It has been suggested that males and females might react differently to treatment with phytochemicals [48,49,50].

In summary, the results of our study suggest that BCP is capable of attenuating an early step in atherogenesis, namely the oxidation of LDL. Atherosclerosis can readily result in cardiovascular disease, which has become epidemic and is a leading cause of death globally [51]. Our findings could encourage the implementation of prospective clinical studies, especially involving humans, to assess the possible atheroprotective and thus health-promoting benefits of BCP.

## 5. Conclusions

We show herein that Beta-Caryophyllene (BCP), a compound present in numerous plants and food, inhibits copper-mediated oxidation of LDL isolates in vitro in a concentration-dependent manner at concentrations ranging from 0 to 150 µg/mL. Quantum chemical calculations show that BCP exerts its anti-oxidant effect probably by its ability to scavenge hydroxyl radicals, hydroperoxyl radicals and hydrogen peroxide. BCP could therefore be an excellent candidate for impeding oxidative stress and inflammation.

## Figures and Tables

**Table 1:** Gibbs free energies of reaction. Negative values of Gibbs free energy indicate exergonic reactions, i.e., abstraction of one hydrogen atom from BCP. Positive values denote endergonic reactions.

Radical	Position of H-Abstraction	Gibbs Free Energy [kcal/mol]
	3	−26.54
	10	−33.60
Hydroxyl radical	15	−32.56
	25	−24.97
	30	−34.26
	3	06.95
	10	−00.11
Hydroperoxyl radical	15	00.92
	25	08.52
	30	−00.77
	3	22.72
	10	15.66
Superoxide radical anion	15	16.69
	25	24.29
	30	15.00

## Data Availability

The raw data supporting the conclusions of this article will be made available by the authors on request.

## Abbreviations

The following abbreviations are used in this manuscript:

Apo B	Apolipoprotein B
BCP	Beta-Caryophyllene
CB	Cannabinoid receptors
DNPH	2,4-dinitrophenylhydrazine
EA.hy926	A human vascular endothelial cell line
HO^•^	Hydroxyl radical
HOO^•^	Hydroperoxyl radical
LDL	Low-density lipoprotein
LPO	Lipid hydroperoxides
MDA	malondialdehyde
MTT	3-(4,5-dimethyl-2-thiazolyl)-2,5-diphenyl-2H-tetrazolium bromide
nLDL	Native low-density lipoprotein
O_2_^•−^	Superoxide radical anion
oxLDL	Oxidatively modified low-density lipoprotein
ROS	Reactive oxygen species
